# Clinical Profile and Dental Treatment Needs of Children with Special Healthcare Needs Undergoing General Anaesthesia: A Retrospective Study

**DOI:** 10.3390/children13081034

**Published:** 2026-08-03

**Authors:** María Carmona-Santamaría, María Isidora Sarciat Aguayo, Laura Marqués-Martínez, Juan Ignacio Aura-Tormos, Clara Guinot-Barona, Esther García-Miralles

**Affiliations:** 1 Departamento de Odontología, Facultad de Medicina y Ciencias de la Salud, Universidad Católica de Valencia San Vicente Mártir, 46001 Valencia, Spain; maria.carmona@ucv.es (M.C.-S.); masara@mail.ucv.es (M.I.S.A.); laura.marques@ucv.es (L.M.-M.); 2 Departamento d’Estomatologia, Facultad de Medicina i Odontologia, Universidad de València, 46010 Valencia, Spain; juan.aura@uv.es (J.I.A.-T.); m.esther.garcia@uv.es (E.G.-M.)

**Keywords:** special healthcare needs, general anaesthesia, paediatric dentistry, autism spectrum disorder, dental caries, hospital dentistry, retrospective study

## Abstract

**Highlights:**

**What are the main findings?**

- ASD was the leading systemic condition in both the outpatient clinic and the subgroup treated under general anaesthesia (outpatient: 25.0%; GA subgroup: 42.9%), a significantly higher prevalence compared with a non-overlapping outpatient-only comparator (*p* = 0.003); 81.0% of GA patients required two or more procedure types per session (mean 2.5 ± 1.0).
- Dental caries (61.0%) and root remnants (32.1%) far exceeded general Spanish population rates, indicating a critical preventive deficit in CSHCN.

**What are the implications of the main findings?**

- Referrals originated from 27 hospitals across the Valencian Community, highlighting the regional referral role of the centre.
- Caregiver-targeted preventive programmes and expanded hospital dental capacity may reduce reliance on resource-intensive surgical intervention in this population.

**Abstract:**

**Background/Objectives:** Children with special healthcare needs (CSHCN) face significant barriers to conventional dental care and frequently require general anaesthesia (GA). Data characterising the clinical and therapeutic profile of this population in the Spanish public health system remain scarce. This study aimed to describe the systemic conditions, oral pathology, and dental procedures performed in CSHCN attending a tertiary-care paediatric oral and maxillofacial surgery service, and to characterise the subgroup of patients requiring treatment under GA relative to the wider clinic population. **Methods:** A retrospective, descriptive, observational study was conducted at the Hospital Universitario y Politécnico La Fe (Valencia, Spain) from November 2022 to February 2026. Data were extracted from electronic health records for 160 outpatient clinic patients aged 0–14 years (70.6% male), of whom 42—a subgroup, not an independent sample—subsequently underwent dental treatment under GA (76.2% male). Sex, age, and autism spectrum disorder (ASD) status were compared between the GA subgroup and a non-overlapping outpatient-only comparator (n = 118) using Fisher’s exact test and Welch’s *t*-test. **Results:** ASD was the most prevalent systemic condition in both the outpatient clinic (25.0%) and the GA subgroup (42.9%); a statistically significant association with ASD status was observed against the non-overlapping outpatient-only comparator (18.6% ASD; *p* = 0.003), while sex (*p* = 0.432) and age (*p* = 1.00) did not differ. Dental caries was the most common finding at outpatient assessment (61.0%), followed by root remnants (32.1%). Among GA patients, tooth extraction was performed in 78.6%, composite restoration in 71.4%, and professional dental cleaning in 50.0%. The mean number of procedure types per GA patient was 2.5 ± 1.0 (range 1–5); 81.0% received two or more procedure types in a single session. **Conclusions:** CSHCN attending this service present advanced oral disease, and those referred for GA had a significantly higher prevalence of ASD than the remaining clinic population, requiring complex, multi-procedure interventions in a single session. The wide geographic pattern of referrals suggests that further evaluation of the distribution of hospital dental services within the public health network may be warranted, and caregiver-targeted preventive programmes may represent a promising strategy to reduce the oral disease burden in this population.

## 1. Introduction

Paediatric patients with special healthcare needs (CSHCN) are defined as children under 18 years of age who have any physical, developmental, mental, sensory, behavioural, cognitive, or emotional condition that requires medical treatment, health interventions, or specialised programmes [1]. These conditions—whether congenital, acquired, or resulting from trauma—frequently limit daily self-care activities and predispose affected children to a higher burden of systemic and oral disease [2].

The CSHCN category encompasses a broad and heterogeneous range of conditions, including neurodevelopmental disorders (autism spectrum disorder [ASD], attention-deficit/hyperactivity disorder, and intellectual disability), motor disorders such as cerebral palsy, seizure disorders, genetic syndromes, and complex chronic systemic disease (e.g., congenital heart disease, renal disease). Each of these categories affects oral health through partially distinct mechanisms: neurodevelopmental and behavioural conditions primarily limit cooperation with oral hygiene and dental examination and are frequently associated with sensory hypersensitivity that makes toothbrushing aversive; motor disorders can impair the manual dexterity required for effective self-performed hygiene and may be accompanied by orofacial dysfunction affecting mastication and clearance of food debris; and chronic systemic disease is frequently accompanied by long-term polypharmacy with xerostomic side effects, which reduces salivary protection against caries [3].

Oral health in CSHCN is consistently poorer than in the general paediatric population. Contributing factors include difficulties coordinating oral hygiene with broader medical management, polypharmacy with xerostomic side effects, high-sugar dietary preferences, limited cooperation for conventional dental procedures, and insufficient dental-health education among caregivers [3]. As a result, rates of dental caries, gingivitis, and periodontal disease are substantially elevated in this group [4].

When the extent of oral disease, the patient’s medical complexity, or behavioural limitations preclude management under local anaesthesia alone, general anaesthesia (GA) becomes the only viable option to deliver safe, effective, and comprehensive dental care in a single session [5,6]. Despite its clinical necessity, GA-based dental treatment is resource-intensive, requiring specialised anaesthetic and surgical teams, dedicated theatre time, and hospitalisation infrastructure; as a result, provision remains limited and geographically unequal within Spain’s decentralised public health system [7].

The existing literature on the profile of CSHCN receiving dental treatment under GA is predominantly drawn from Northern European, North American, and East Asian settings [2,8,9]. Spanish and Valencian-specific data are particularly scarce; existing single-centre reports include an early Valencian series [10], together with more detailed studies from Murcia [11] and Madrid [12,13]; comparable single-centre data from elsewhere in Europe, such as a recent Croatian series, are similarly limited in scope [14]. Detailed characterisation of the systemic conditions, oral pathology burden, and procedures performed in these patients is essential to inform resource allocation and advocate for equitable expansion of hospital dental services.

The Hospital Universitario y Politécnico La Fe (Valencia, Spain) operates one of the few publicly funded Paediatric Oral and Maxillofacial Surgery services in the Valencian Community, receiving referrals from hospitals throughout the region. This study aimed to characterise the clinical and therapeutic profile of CSHCN attending this service from 2022 to 2026, with three specific objectives: (i) to describe demographic and systemic characteristics of the outpatient clinic population and of the subgroup subsequently referred for treatment under GA; (ii) to quantify oral pathology findings in the outpatient clinic population and dental procedures performed under GA, including procedure intensity per patient; and (iii) to formally compare sex, age, and ASD status between the GA subgroup and a non-overlapping outpatient-only comparator group.

## 2. Materials and Methods

### 2.1. Study Design

A retrospective, descriptive, observational study was conducted at the Paediatric Oral and Maxillofacial Surgery Service of the Hospital Universitario y Politécnico La Fe, Valencia, Spain, covering the period from 2 November 2022 to 5 February 2026. 

### 2.2. Ethics Approval

The study was approved by the Clinical Research Ethics Committee (CEIC) of the Hospital Universitario y Politécnico La Fe, Valencia, with registration number 2026-0308-1 (protocol code ODP-QLAFE), approved 23 June 2026. The retrospective, non-interventional design based exclusively on anonymised clinical records met the criteria for a waiver of individual informed consent, as approved by the CEIC. 

### 2.3. Study Population and Eligibility Criteria

**Figure 1:**
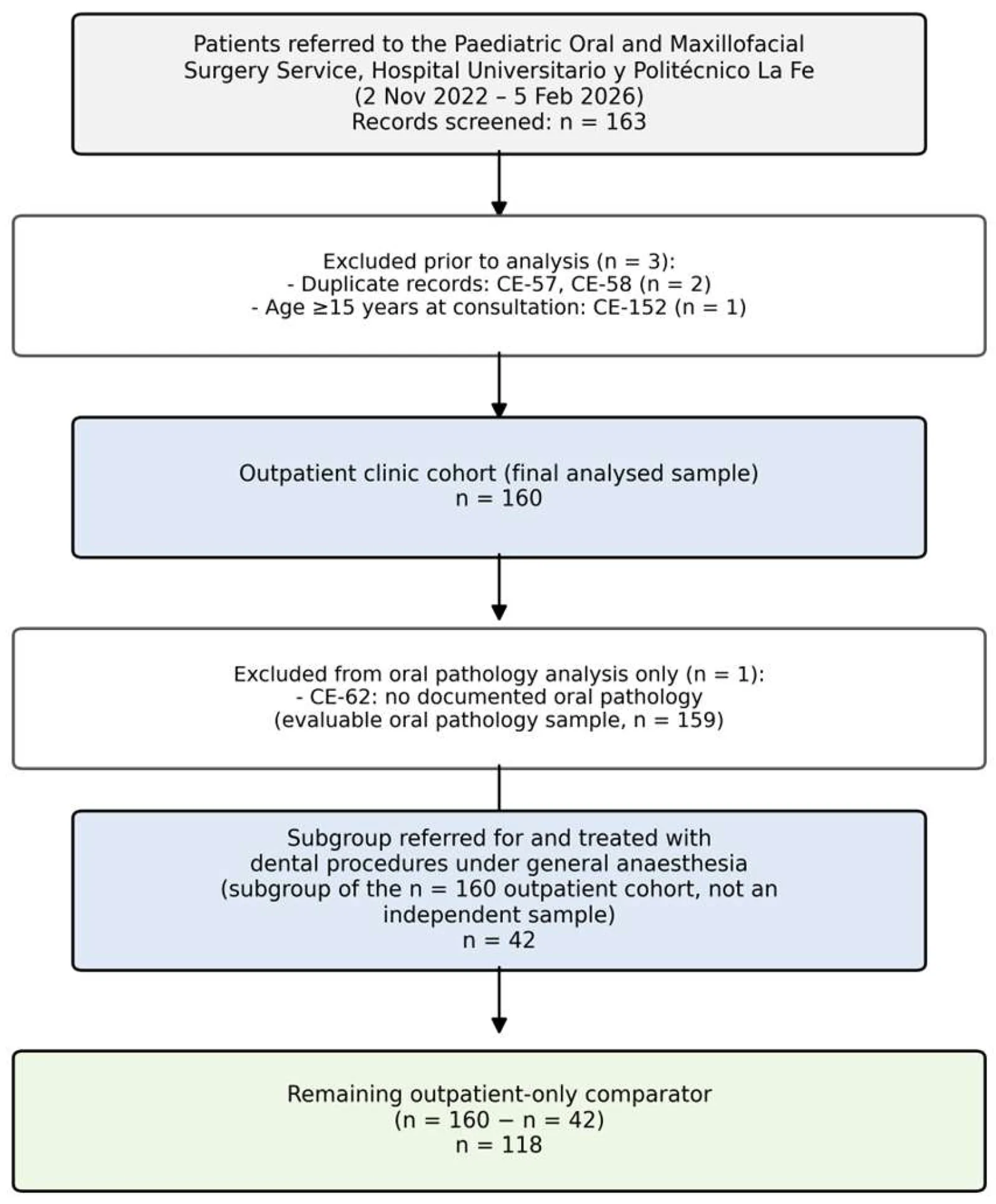
Patient flow through the study.

Patients were eligible for inclusion if they (i) were aged 0–14 years at the time of the appointment, (ii) had been referred by a primary or secondary care centre, (iii) had a documented disability, chronic systemic condition, or severe behavioural management problem, and (iv) resided in the Valencian Community. The 0–14-year age range was applied because it corresponds to the age band covered by the paediatric dental referral pathway through which patients reach this service; patients aged 15 years and older in the Valencian Community are managed through adult hospital dental and oral surgery pathways rather than the paediatric referral circuit analysed here, so extending the sample beyond 14 years would have mixed two organisationally distinct referral populations, a restriction we acknowledge limits generalisability to adolescents aged 15–17 (the Limitations Section). Exclusion criteria were age ≥ 15 years, American Society of Anesthesiologists (ASA) physical status classification IV or higher, and oral pathology outside the publicly funded dental care portfolio of the Valencian Health System. Three patients were excluded prior to analysis: two duplicate records (CE-57 and CE-58, confirmed by chart review) and one patient aged 15 years at the time of consultation (CE-152), yielding a final outpatient sample of 160 patients (Figure 1). One additional outpatient record (CE-62) had no documented oral pathology and was excluded from the oral pathology frequency analysis (evaluable n = 159). Of these 160 outpatient clinic patients, 42 were, following clinical assessment, referred for and underwent dental treatment under general anaesthesia due to the extent of disease, patient complexity, or inability to cooperate for conventional chairside treatment. This GA group is therefore a subgroup of, and not an independent sample from, the 160-patient outpatient cohort: all 42 GA patients are also outpatient clinic patients, and any statistical comparison between the two must account for this nesting (Section 2.5).

### 2.4. Data Collection

Data were extracted from the hospital’s electronic health records system and from surgical register logbooks. Each patient was assigned a unique alphanumeric code to ensure anonymisation. Associated systemic conditions were as documented by the referring medical or paediatric specialist in the referral letter and hospital record; oral pathology was assessed clinically by the paediatric dentists of the Oral and Maxillofacial Surgery Service at the outpatient visit, using descriptive diagnostic categories rather than standardised indices (e.g., ICDAS, plaque index, or gingival index), which were not part of the routine charting protocol during the study period and could therefore not be analysed retrospectively; a separate inter-examiner calibration exercise for this retrospective analysis was not performed, which we acknowledge as a limitation (the Limitations Section). Variables recorded for outpatient clinic patients included: patient code, sex, age, referring hospital, date of consultation, associated systemic condition(s), and oral pathology diagnosed. For patients undergoing dental treatment under GA, additional variables recorded were the surgical date and all dental procedures performed (tooth-by-tooth, using the FDI two-digit notation system). Procedure intensity was defined operationally as the number of distinct procedure types (extraction, restoration, cleaning, provisional resin restoration, fissure sealant, fluoride application) performed on a patient within a single GA session, counted as a simple integer; this is a descriptive proxy for surgical complexity and not a validated severity instrument.

### 2.5. Statistical Analysis

Descriptive statistics were calculated for all variables. Categorical variables are presented as absolute frequencies (n) and relative frequencies (%), with 95% confidence intervals (CI) reported for the principal proportions. Continuous variables (age, procedures per patient) are reported as mean ± standard deviation (SD), median, and range. As patients may present with more than one systemic condition and/or more than one oral finding, frequencies for these variables may exceed 100% and are calculated over the respective cohort totals. Because the 42 patients treated under GA are a subgroup of, rather than independent from, the 160-patient outpatient cohort (Section 2.3), standard independent-samples inferential tests cannot be applied directly between the GA subgroup and the full outpatient cohort. To obtain a statistically independent comparison, we constructed an outpatient-only comparator (n = 118) by excluding the 42 GA patients from the outpatient cohort and recalculating the descriptive statistics for the remaining 118 patients. Fisher’s exact test was used to compare sex and ASD status, and Welch’s *t*-test was used to compare age, between the GA subgroup and this outpatient-only comparator; these three comparisons were the only inferential tests performed.

Multivariate regression was not performed, given the limited size of the GA subgroup (n = 42) relative to the number of candidate predictors (sex, age, disability type, and referring hospital, the latter comprising 27 categories), which would risk over-fitting. All analyses were performed using IBM SPSS Statistics, version 29.0 (IBM Corp., Armonk, NY, USA) and 3.11 (SciPy 1.11), with a two-sided significance threshold of *p* < 0.05.

## 3. Results

### 3.1. Demographic Characteristics

A total of 163 patient records were screened; three were excluded prior to analysis (Figure 1), yielding a final outpatient clinic cohort of 160 patients, of whom 42 subsequently underwent dental treatment under general anaesthesia as a subgroup of this cohort—not an independent sample. Table 1 and Table 2 summarise demographic characteristics and referral patterns.

In the outpatient clinic cohort, 113 patients (70.6%, 95% CI 63.2–77.1%) were male and 47 (29.4%) were female, with a mean age of 9.0 ± 3.1 years (median 9.0, range 0–14). Within the GA subgroup, 32 (76.2%, 95% CI 61.5–86.5%) were male and 10 (23.8%) were female, with a mean age of 9.0 ± 3.1 years (median 9.0, range 0–14). When the GA subgroup was compared against the non-overlapping outpatient-only comparator (n = 118; 68.6% male), the difference in sex distribution was not statistically significant (Fisher’s exact test, OR = 1.46, *p* = 0.432), nor was the difference in age (Welch’s *t*-test, *p* = 1.00; outpatient-only comparator mean age 9.0 ± 3.1 years). Hospital La Fe Campanar was the principal referring centre for both the outpatient cohort and the GA subgroup, accounting for 36 (22.5%) outpatient referrals and 14 (33.3%) of GA referrals. Other major sources of referral were Hospital Arnau de Vilanova (n = 24, 15.0%) and Hospital General de Elche (n = 14, 8.8%). Referrals originated from 27 different hospitals across the Valencian Community.

### 3.2. Systemic Conditions

Table 2 shows the distribution of systemic conditions in the outpatient clinic cohort (n = 160). ASD was the most prevalent condition, documented in 40 patients (25.0%, 95% CI 18.9–32.2%), either in isolation or combined with other diagnoses such as epilepsy or ADHD. Neurodevelopmental delay and psychomotor delay constituted the second most frequent category (n = 17, 10.6%), followed by epilepsy (n = 13, 8.1%) and ADHD (n = 12, 7.5%). Cerebral palsy or spastic tetraparesis was documented in eight patients (5.0%) and congenital heart disease in a further eight (5.0%). In total, neurodevelopmental conditions (ASD, ADHD, neurodevelopmental delay, cerebral palsy) accounted for 82 patients (51.3%) of the outpatient cohort.

**Figure 2:**
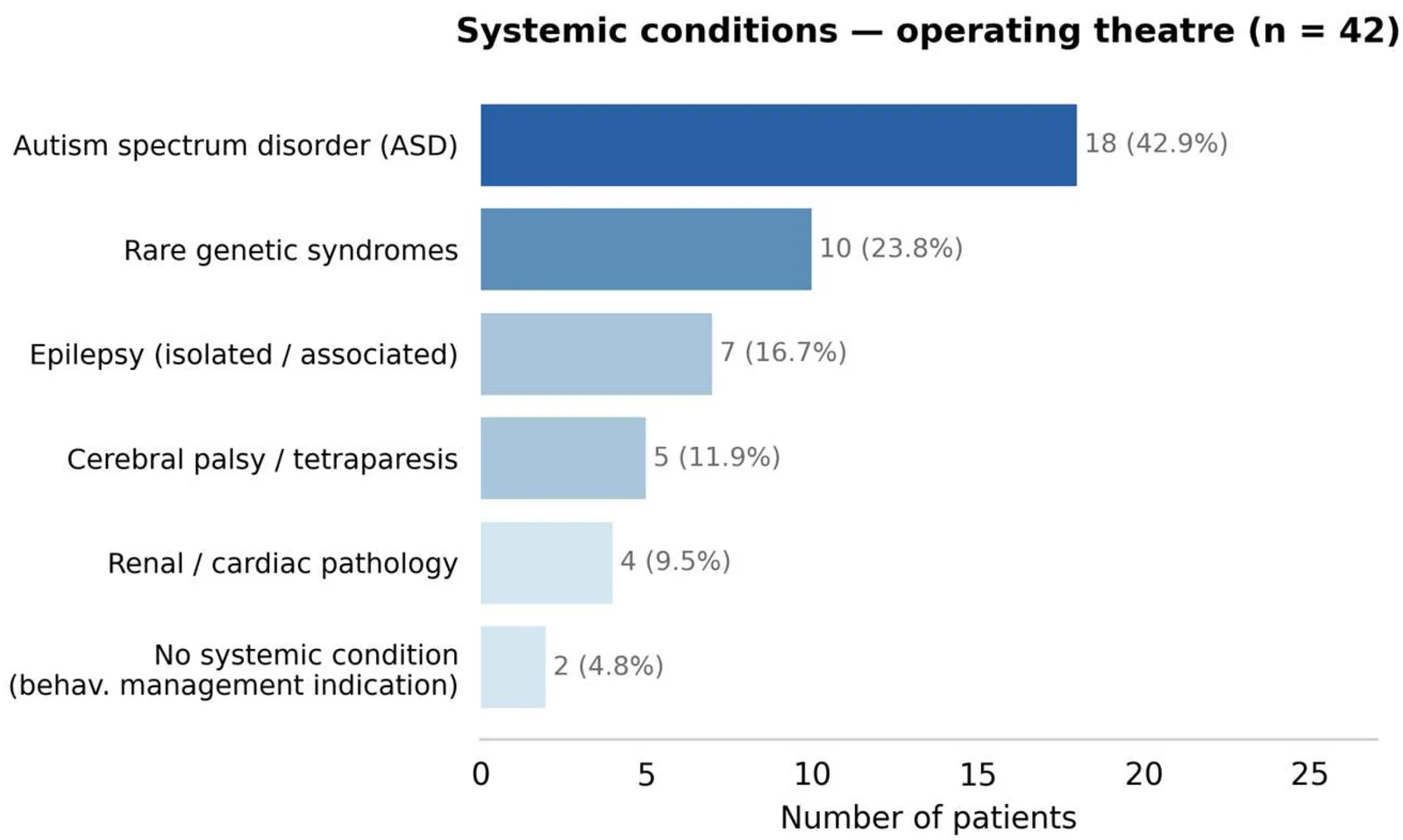
Distribution of systemic conditions among patients treated under general anaesthesia (n = 42).

Within the GA subgroup, ASD (alone or in combination) was present in 18 of 42 patients (42.9%, 95% CI 29.1–57.8%). Compared against the non-overlapping outpatient-only comparator (22/118, 18.6%, 95% CI 12.6–26.6%), patients referred for treatment under GA had significantly higher odds of ASD (Fisher’s exact test, OR = 3.27, *p* = 0.003). Rare genetic syndromes or complex multisystem conditions were documented in ten GA patients (23.8%), followed by epilepsy in seven (16.7%) and cerebral palsy or tetraparesis in five (11.9%) (Figure 2). Neurodevelopmental conditions overall (ASD, ADHD, neurodevelopmental delay, cerebral palsy) accounted for 30 of 42 GA patients (71.4%), compared with 52 of the 118 outpatient-only patients (44.1%); this broader category was reported descriptively and was not among the three pre-specified inferential tests.

### 3.3. Oral Pathology

Table 3 summarises oral pathology findings identified at outpatient clinic assessment (n = 159 evaluable; one record excluded due to missing data). Outpatient clinic visits in this dataset represent diagnostic and triage consultations at which systemic condition, oral pathology, and treatment need were assessed; specific chairside treatments delivered during these visits, if any, were not systematically coded as a separate variable in the retrospective database and are therefore not reported here. Dental caries was the predominant finding, affecting 97 patients (61.0%, 95% CI 53.3–68.2%). Root remnants—indicative of advanced or untreated carious lesions—were present in 51 patients (32.1%, 95% CI 25.3–39.7%). Dental calculus was documented in 16 patients (10.1%). Twenty-seven patients (17.0%) presented with no identifiable oral pathology. Gingival inflammation (combining gingivitis, gingival hyperplasia, and hypertrophy) was noted in 10 patients (6.3%); as periodontal indices (plaque index, gingival index) were not part of the routine charting protocol, this finding reflects a general descriptive diagnosis rather than a standardised periodontal score. Molar–incisor hypomineralisation (MIH) was identified in three patients (1.9%).

### 3.4. Dental Procedures Under General Anaesthesia and Procedure Intensity

Table 3 also summarises dental procedures performed within the GA subgroup (n = 42). Tooth extraction was performed in 33 patients (78.6%, 95% CI 64.1–88.3%), composite or glass-ionomer restoration in 30 (71.4%, 95% CI 56.4–82.8%), professional dental cleaning in 21 (50.0%, 95% CI 35.5–64.5%), provisional resin restoration (PRR) in nine (21.4%), fissure sealant in four (9.5%), and topical fluoride application in four (9.5%). Only four patients (9.5%) received exclusively preventive procedures (cleaning and/or fluoride application without any restorative or surgical intervention).

The mean number of procedure types per patient was 2.5 ± 1.0 (median 2.5, range 1–5). Thirty-four patients (81.0%, 95% CI 66.7–90.0%) received at least two different procedure types in a single GA session, and 21 (50.0%) received three or more. Twenty-six patients (61.9%) underwent simultaneous extraction and restoration, confirming the complex, multi-modal nature of these surgical episodes (Table 4).

## 4. Discussion

This retrospective study describes the clinical and therapeutic profile of 160 CSHCN seen at the outpatient dental clinic of a tertiary-care public hospital in Valencia, Spain, over 39 months, including detailed characterisation of the subgroup of 42 patients who underwent dental treatment under general anaesthesia within this cohort. The principal findings are (i) ASD accounted for a significantly greater proportion of patients within the GA subgroup than among the remaining outpatient-only patients; (ii) dental caries and root remnants dominated the oral disease burden identified at outpatient assessment; and (iii) GA sessions were characterised by high procedural complexity, with 81.0% of patients receiving multiple simultaneous intervention types and a mean of 2.5 procedure types per patient.

ASD was the most prevalent systemic condition in both the outpatient clinic (25.0%) and the GA subgroup (42.9%). This predominance is consistent with international literature: Hieronymus et al. reported ASD as the leading indication for GA dentistry in a 10-year German retrospective study [15], a pattern corroborated by a more recent German tertiary-hospital series [16], and Alfarraj et al. identified neurodevelopmental disorders as the primary referral indication in a tertiary Saudi centre [9]. Children with ASD present profound communication barriers, sensory hypersensitivity, and intolerance of disrupted routines, all of which render conventional chairside management frequently impossible beyond limited preventive measures [17]. Against the non-overlapping outpatient-only comparator, the GA subgroup had significantly higher odds of ASD (42.9% vs. 18.6%; OR = 3.27, *p* = 0.003)—a pattern consistent with, and numerically stronger than, the descriptive difference observed against the full, non-independent outpatient cohort, and likely reflecting the greater difficulty of providing chairside treatment for children with ASD. Neurodevelopmental conditions overall (ASD, ADHD, neurodevelopmental delay, cerebral palsy) accounted for 71.4% of GA patients but only 44.1% of the outpatient-only comparator group; this broader category was reported descriptively only.

Male predominance was observed both in the outpatient clinic (70.6%) and within the GA subgroup (76.2%), consistent with the well-established higher prevalence of ASD and other neurodevelopmental disorders in males [15,18]. Unlike ASD status, sex distribution did not differ between the GA subgroup and the outpatient-only comparator (68.6% male; OR = 1.46, *p* = 0.432), suggesting it does not clearly differentiate patients requiring GA from the wider clinic population; the higher male proportion in the GA subgroup may nonetheless partly reflect its greater proportion of ASD patients.

Dental caries was the most prevalent oral finding among outpatient clinic patients (61.0%), and root remnants—indicative of advanced or neglected carious lesions—were present in nearly one-third (32.1%). These figures substantially exceed national prevalence data from the Encuesta de Salud Oral en España 2020 [19], which reported caries prevalence of 35.5% in 5–6-year-old Spanish children and 30.1% at age 12, and align with the systematic review by Pecci-Lloret et al., which documented a markedly elevated caries burden in CSHCN compared with the general paediatric population [20], as well as with a Spanish single-centre series describing similarly high treatment needs among CSHCN referred for GA [11]. Contributing factors include xerostomic medications (anticonvulsants, antipsychotics), dietary selectivity, inadequate toothbrushing due to oral hypersensitivity or carer burden, and infrequent preventive dental visits [21]. Anwar et al. demonstrated that family-level factors—parental knowledge, brushing supervision, and dietary habits—are independently associated with caries severity in children with special needs [3].

Tooth extraction was the most common procedure under GA (78.6%), reflecting advanced disease stage at surgical referral and consistent with findings from Alfarraj et al. [9] and Ciftci and Yazicioglu [2]. The simultaneous high rate of composite restoration (71.4%) indicates that clinicians prioritised tooth preservation wherever feasible. The high frequency of PRR (21.4%) reflects the intention to maintain pulpal vitality in primary molars. The finding that 81.0% of patients required two or more procedure types and 61.9% required simultaneous extraction and restoration in a single GA session underscores the complexity of disease at the time of surgical referral and suggests accumulated dental need that could not be addressed through incremental chairside visits. Only 9.5% of GA patients received exclusively preventive treatment, confirming that GA is predominantly reserved for complex restorative-surgical cases in this service.

The wide geographic breadth of referrals—from 27 different hospitals spanning from Castellón to Orihuela—with Hospital La Fe Campanar as the leading referrer (22.5% outpatient; 33.3% GA), may reflect both proximity and established inter-hospital referral pathways, consistent with the tertiary reference role documented by Nikolovski et al. for congenital heart disease patients [22]. As this pattern is drawn from a single tertiary-referral centre, it should be interpreted as descriptive of this centre’s catchment rather than as direct evidence of unmet need across the wider Valencian public health network; any implications for resource allocation should therefore be regarded as hypothesis-generating rather than conclusive, and causal inferences cannot be drawn from this single-centre retrospective design. Al-Mashhadani et al. identified limited availability of specialised services and insufficient trained personnel as key barriers to dental care access for CSHCN more broadly [21].

Dental cleaning was performed in half of all GA patients (50.0%), often as a component of multi-procedure sessions, highlighting the accumulated preventive deficit in this population. Yilmaz et al. demonstrated that GA-based dental treatment significantly improves oral health-related quality of life in children who cannot otherwise access adequate care [23], supporting the continued development of hospital-based services; oral health-related quality of life was not itself measured in the present retrospective dataset. A recent systematic review by Carmona-Santamaría et al., conducted within the same clinical network, characterised the evidence base for GA utilisation patterns in paediatric dentistry [6]; the present study adds granular, real-world data profiling both the outpatient clinic population and the GA subgroup within it, across a geographically defined referral network.

### Limitations

This study has several limitations:

- Retrospective, single-centre design, which limits generalisability and precludes causal inference; as referrals came exclusively from one tertiary service, the cohort likely over-represents more complex cases (referral bias).
- The GA subgroup is nested within the outpatient cohort; we corrected for this in the three formally tested variables (sex, age, ASD status) using a non-overlapping comparator, but did not extend this correction to other, descriptively reported variables, and applied no multiple-comparisons adjustment.
- Multivariate regression was not performed, given the limited GA subgroup size (n = 42) relative to the number of candidate predictors, which would risk model over-fitting.
- Standardised oral health indices (ICDAS, plaque index, gingival index), graded systemic severity, behavioural cooperation, and socioeconomic variables were not recorded in the retrospective database and could not be analysed.
- No postoperative follow-up data (complications, reinterventions, caries recurrence, or oral health-related quality of life) were captured; the study reflects a single time point.
- One outpatient record was excluded from the oral pathology analysis for missing data, and three patients were excluded for protocol deviations (duplicates and age non-compliance).
- Systemic condition categorisation relied on non-standardised clinical record terminology, and inter-examiner reliability of oral pathology diagnosis was not formally assessed.
- The 0–14-year age restriction limits generalisability to adolescents aged 15–17 managed through adult referral pathways.

Future prospective, multicentre studies with standardised diagnostic indices, socioeconomic data, and longitudinal follow-up are needed to further characterise this population.

## 5. Conclusions

This study characterises the clinical and therapeutic profile of CSHCN attending a tertiary-care hospital dental service in Valencia, Spain, over 39 months, including the subgroup of patients treated under general anaesthesia within this cohort. ASD was the leading systemic condition overall and was significantly more frequent among patients requiring treatment under GA than among the remaining outpatient-only patients; dental caries and root remnants constituted the dominant oral disease burden identified at outpatient assessment. GA sessions were characterised by high procedural complexity, with most patients receiving multiple simultaneous intervention types.

These findings suggest that many patients presented with advanced oral disease requiring complex, multi-procedure intervention. Based on the referral pattern observed, spanning 27 hospitals across the Valencian Community to a single tertiary centre, we suggest that further evaluation of the distribution of hospital dental services within the public health network may be warranted, while recognising that this single-centre retrospective design cannot establish need or resource allocation directly. Caregiver-targeted preventive programmes may represent a promising strategy to help reduce the oral disease burden in CSHCN and, potentially, the need for complex GA-based intervention; this remains a suggestion arising from the findings rather than a direct result of the study.

## Figures and Tables

**Table 1:** Demographic and referral characteristics of the study cohorts.

Variable	Outpatient Clinic (n = 160)	GA Subgroup (n = 42)
**Sex**		
Male, n (%)	113 (70.6%)	32 (76.2%)
Female, n (%)	47 (29.4%)	10 (23.8%)
Age (years), mean ± SD	9.0 ± 3.1	9.0 ± 3.1
Median (range)	9.0 (0–14)	9.0 (0–14)
Main referring hospital, n (%)		
H. La Fe Campanar	36 (22.5%)	14 (33.3%)
H. Arnau de Vilanova	24 (15.0%)	4 (9.5%)
H. General de Elche	14 (8.8%)	7 (16.7%)
H. General de Valencia	12 (7.5%)	1 (2.4%)
H. de Sagunto	8 (5.0%)	—
Other hospitals	66 (41.2%)	16 (38.1%)

Referrals originated from 27 hospitals across the Valencian Community.

**Table 2:** Distribution of systemic conditions in the outpatient cohort (n = 160).

Systemic Condition	n	% *
Autism spectrum disorder (ASD)	40	25.0%
Neurodevelopmental delay/psychomotor delay	17	10.6%
Epilepsy (isolated or associated)	13	8.1%
ADHD	12	7.5%
Cerebral palsy/spastic tetraparesis	8	5.0%
Congenital heart disease/cardiac pathology	8	5.0%
Cleft lip/palate	4	2.5%
Renal pathology (chronic/transplant)	4	2.5%
Tuberous sclerosis	4	2.5%
Down syndrome	3	1.9%
Ectodermal dysplasia	3	1.9%
Mucopolysaccharidosis/lysosomal storage disease	3	1.9%
Other conditions **	37	23.1%
No systemic condition (behavioural management indication)	10	6.3%
**Total**	**160**	**100%**

* Patients may carry more than one diagnosis. ** Other conditions included rare genetic syndromes, metabolic disorders, neuromuscular diseases, and miscellaneous medical conditions, each occurring in fewer than three patients.

**Table 3:** Oral pathology (outpatient clinic) and dental procedures performed under general anaesthesia (operating theatre).

Oral Pathology—Outpatient Clinic (n = 159) ^†^	n	% ^‡^
Dental caries	97	61.0%
Root remnants	51	32.1%
Dental calculus (tartar)	16	10.1%
No oral pathology	27	17.0%
Gingival inflammation (gingivitis/hyperplasia/hypertrophy)	10	6.3%
Dental agenesis	5	3.1%
Molar–incisor hypomineralisation (MIH)	3	1.9%
Bruxism	3	1.9%
Other findings	12	7.5%
**Dental Procedures—Operating Theatre (n = 42)**	**n**	**%**
Extraction	33	78.6%
Composite/GIC restoration	30	71.4%
Professional dental cleaning	21	50.0%
Provisional resin restoration (PRR)	9	21.4%
Fissure sealant	4	9.5%
Fluoride application	4	9.5%

^†^ One record (CE-62) with missing oral pathology data excluded. ^‡^ Percentages exceed 100% as patients may present with/receive more than one condition/procedure.

**Table 4:** Comparative characteristics of the GA subgroup and the non-overlapping outpatient-only comparator group (n = 118). Only sex, age, and ASD status were formally tested; remaining rows are descriptive.

Characteristic	GA Subgroup (n = 42)	Outpatient-Only (n = 118)	*p*-Value
Age (years), mean ± SD	9.0 ± 3.1	9.0 ± 3.1	1.000
Male sex, n (%)	32 (76.2%)	81 (68.6%)	0.432
ASD, n (%)	18 (42.9%)	22 (18.6%)	0.003 *
Neurodevelopmental conditions total, n (%) ^†^	30 (71.4%)	52 (44.1%)	—
Mean procedure types per GA patient (range)	2.5 ± 1.0 (1–5)	—	—
Patients receiving ≥2 procedure types, n (%)	34 (81.0%)	—	—
Patients receiving extraction + restoration combined, n (%)	26 (61.9%)	—	—

* Statistically significant (*p* < 0.05). ^†^ Neurodevelopmental conditions include autism spectrum disorder (ASD), attention-deficit/hyperactivity disorder (ADHD), neurodevelopmental delay, and cerebral palsy. This variable is presented descriptively only and was not included among the pre-specified inferential comparisons. — Not applicable.

## Data Availability

The data that support the findings of this study are available from the corresponding author upon reasonable request, subject to applicable privacy regulations.

## Abbreviations

The following abbreviations are used in this manuscript:

CSHCN	Children with special healthcare needs
GA	General anaesthesia
ASD	Autism spectrum disorder
ADHD	Attention deficit hyperactivity disorder
MIH	Molar–incisor hypomineralisation
PRR	Provisional resin restoration
FDI	Fédération Dentaire Internationale
CEIC	Comité de Ética de Investigación Clínica
GDPR	General Data Protection Regulation
SD	Standard deviation
CI	Confidence interval
OR	Odds ratio
ICDAS	International Caries Detection and Assessment System
UV	Universitat de València
UCV	Universidad Católica de Valencia
